# Noroviral P-Particles as an *In Vitro* Model to Assess the Interactions of Noroviruses with Probiotics

**DOI:** 10.1371/journal.pone.0089586

**Published:** 2014-02-21

**Authors:** Antonio Rubio-del-Campo, José M. Coll-Marqués, María J. Yebra, Javier Buesa, Gaspar Pérez-Martínez, Vicente Monedero, Jesús Rodríguez-Díaz

**Affiliations:** 1 Laboratory of Lactic Acid Bacteria and Probiotics, Biotechnology Department, Institute of Agrochemistry and Food Technology (IATA-CSIC), Valencia, Spain; 2 Department of Microbiology and Ecology, Faculty of Medicine, University of Valencia, Valencia, Spain; Tulane University, United States of America

## Abstract

Noroviruses (NoVs) are the main etiologic agents of acute epidemic gastroenteritis and probiotic bacteria have been reported to exert a positive effect on viral diarrhea. The protruding (P) domain from NoVs VP1 capsid protein has the ability to assemble into the so-called P-particles, which retain the binding ability to host receptors. We purified the P-domains from NoVs genotypes GI.1 and GII.4 as 6X(His)-tagged proteins and determined that, similar to native domains, they were structured into P-particles that were functional in the recognition of the specific glycoconjugated receptors, as established by surface plasmon resonance experiments. We showed that several lactic acid bacteria (probiotic and non-probiotic) and a Gram-negative probiotic strain have the ability to bind P-particles on their surfaces irrespective of their probiotic status. The binding of P-particles (GI.1) to HT-29 cells in the presence of selected strains showed that bacteria can inhibit P-particle attachment in competitive exclusion experiments. However, pre-treatment of cells with bacteria or adding bacteria to cells with already attached P-particles enhanced the retention of the particles. Although direct viral binding and blocking of viral receptors have been postulated as mechanisms of protection against viral infection by probiotic bacteria, these results highlight the need for a careful evaluation of this hypothesis. The work presented here investigates for the first time the probiotic-NoVs-host interactions and points up the NoVs P-particles as useful tools to overcome the absence of *in vitro* cellular models to propagate these viruses.

## Introduction

Noroviruses (NoVs) are members of the *Caliciviridae* family that infect the small intestine and cause the majority of food- and water-borne outbreaks of acute gastroenteritis worldwide, and are also responsible for a significant proportion of sporadic disease, with an unknown prevalence [1]. The incubation time ranges from 15 to 48 hours producing gastroenteritis for 12 to 60 hours. The infection usually courses as a self-limited diarrhea and vomiting but in special cases it can produce severe dehydration and death [2]. NoVs consist of a linear positive-sense single stranded RNA genome of ∼7.7 kb in length, surrounded by a major capsid protein (VP1) of around 500 amino acids. Genetic variability within NoVs is very high. Currently, 5 genogroups exist (GI to GV) that are subdivided into more than 30 genotypes [3]. Genogroups GI and GII represent the major NoVs infecting humans, and the GII.4 genotype has emerged in the last 20 years as the leading NoV strain around the world [4]. Research in the NoVs field has been hampered by the lack of cellular hosts suitable for *in vitro* infection and viral replication [5]. Hence, most research on NoVs biology has been based on the use of virus-like particles (VLPs) produced in the baculovirus system [6]. NoVs recognize human ABH histo-blood group antigens (HBGAs), which are distributed in red blood cells but also in mucosal surfaces of the gastrointestinal tract and act as receptors for virus attachment [7]–[10]. The differences in HBGAs between individuals are also at the basis for host susceptibility to certain human NoVs [11]–[12]. The C-terminal protruding (P)-domain of NoVs VP1 capsid protein, composed of about 300 amino acids, has the ability to auto-assemble into complexes named P-particles, which are structured in twelve P-domain dimers with icosahedral symmetry [13]. P-particles may exhibit the same surface conformation to their parental VLPs, as they have similar antigenic and HBGA-binding profiles. Therefore, they constitute a valuable tool for the study of viral attachment and for the development of vaccines [14].

Lactic acid bacteria (LAB) are microorganisms present in numerous food fermentations and are also normal constituents of the microbiota of the intestinal tract. They have attracted intense interest in the field of functional foods because some LAB strains are probiotics, for which many health claims have been reported. These included protection against harmful intestinal microorganisms and viruses [15]. Numerous clinical trials exist in which evidences have been obtained for a reduction in the duration and severity of viral diarrheas by the oral intake of probiotics [16]–[20]. Enhancement of innate or adaptive immunity has emerged as a mechanism by which probiotics can counteract gastrointestinal viral infections [16], [21]–[25], but it is also postulated that they can interfere with viral infection by competing with binding to viral receptors on the surface of host cells or by direct binding to viral particles, promoting their clearance from the intestine. Certain *Lactobacillus brevis*, *Lactobacillus gasseri*, *Lactobacillus salivarius* and *Lactobacillus acidophillus* strains isolated from human feces are able to bind type-A, -B and -H HBGAs through the lectin-like activity of their surface layer proteins [26]–[27] for which they might compete with NoVs for the attachment sites at the intestinal mucosa. Recently, an *Enterobacter* sp. intestinal isolate was shown to express on its surfaces HBGAs-like substances that allow it to bind NoVs VLPs from different genotypes [28]. Binding between rotavirus and probiotics with clinically demonstrated efficacy in rotavirus diarrhea treatment has also been confirmed [29]. Owing to their relevance in infant viral gastroenteritis, most of the research in the field of viruses and probiotics has been focused on rotaviruses. However, few studies addressed the probiotic–intestinal virus–host interaction [22]–[23], [30]–[31] and no research has been carried out with NoVs. In this work we aimed to develop an *in vitro* model system based on P-particles for testing the likely interaction of NoVs with probiotics and to investigate how probiotics can influence the attachment of NoVs to the host cells.

## Materials and Methods

### Bacterial strains and growth conditions

Eleven probiotic and non-probiotic bacterial strains were assayed in this work: *Escherichia coli* Nissle 1917 (Mutaflor, DSM 6601), *Lactococcus lactis* MG1363 (cheese isolate), *Lactobacillus acidophilus* LA-5 (Chr. Hansen, DSM13241), *Lactobacillus bulgaricus* ATCC11842^T^ (dairy isolate), *Lactobacillus plantarum* 299v (Probi), *L. plantarum* 299v Adh- (an isogenic derivative of 299v strain with decreased adhesion capacities; [32]), *Lactobacillus casei* 431 (Chr. Hansen, ATCC55544), *L. casei* BL23 (CECT5275; [33]), *L. casei* VSL#3 (from the VSL#3 probiotic mix, VSL Pharmaceuticals), *Lactobacillus rhamnosus* GG (intestinal isolate, ATCC53103) and *L. rhamnosus* HN001 (Danisco, SD5675). *Lc. lactis* was grown in M17 medium (Oxoid) supplemented with 0.5% glucose under static conditions at 30°C. *E. coli* Nissle 1917 was grown in LB medium with shaking at 200 rpm and 37°C. All the *Lactobacillus* strains were grown in MRS medium (Difco) at 37°C under static conditions. *E. coli* DH10B was utilized as cloning host and *E. coli* BL21 [pREP4GroES/EL] [34] was utilized for protein expression. These strains were grown in LB medium at 37°C with shaking at 200 rpm. When required, ampicillin was used at 100 µg ml^−1^ and kanamycin was used at 25 µg ml^−1^.

### Expression and purification of NoVs P-particles

The genes coding for the VP1 capsid protein of the NoVs VA387 (GII.4 genotype) and Norwalk (GI.1 genotype) (GeneBank accession numbers AY038600 and M87661, respectively) were synthesized by GeneArt (Life Technologies). The region encoding the P-domain of the VA387 strain was amplified from the synthesized genes with primers p524 (5′-GCACGGATCCTCAAGAACTAAACCATTCACC) and p590 (5′-GCATGCGGCCGC**TTA**GCAAAAGCAATCGCCACGGCAATCGCATAATGCACGTCTGCGCCCCGC). The reverse primer incorporates the bases encoding a cysteine-rich peptide CDCRGDCFC to favor the P-particle stability [13]. To amplify the fragment encoding the Norwalk strain P-domain a modification of the p649 primer (p649-mod: 5′-GCGTGGATCCTGCAACGGCCGTTGCCAGAAAACCAGGCCCTTCACAC), incorporating the CNGRC cysteine-rich domain was used as forward primer and the p494 primer (5′-GGACGCGGCCGC**TTA**TCGGCGCAGACCAAGCCT) was used as the reverse primer [13]. Restriction sites introduced for cloning are underlined and the stop codons are in bold. The PCR amplifications were performed with *Pfx* polymerase (Invitrogen) and the amplified fragments were digested with BamHI and cloned in the pQE80 (Qiagen) vector digested with BamHI and SmaI and transformed into *E. coli* DH10B. Integrity of the sequences was verified by sequencing and the generated plasmids were transferred to *E. coli* BL21 carrying the pREPGroES/EL plasmid [34] coding for *E. coli* chaperones to improve solubility of recombinant P-particles. To further improve recombinant P-particles solubility and recovery, *E. coli* was grown overnight at 25°C and induced with 0.2 mM IPTG during 4 hours at the same temperature. After this, the bacterial cells were harvested by centrifugation and frozen at −80°C. NoVs P-particles were purified using and Äkta Prime FPLC system (GE Healthcare) and 1 ml crude FastFlow Ni-NTA columns (GE Healthcare). The bacterial cells pellets were thawed and resuspended in buffer A (50 mM Tris-HCl pH 7.5, 100 mM NaCl, 50 mM NaSO_4_) supplemented with 0.5 mM dithioerythritol, 0.5 mM phenylmethysulfonyl fluoride and 1 mg/ml lysozyme and incubated for 30 min at room temperature. Cell lysates were obtained by sonication on ice followed by centrifugation at 20.000×*g* for 20 min at 4°C. Clarified lysates were then loaded on the Ni-NTA columns which were washed with buffer A with an imidazole gradient from 0 to 500 mM. For the elution of the P-particles buffer A containing 2 M imidazole was utilized. Fractions containing recombinant proteins were collected and stored at 4°C.

### Functional characterization of the NoVs P-particles

For cross-linking experiments 5 µg of P-particles from GI.1 and GII.4 NoVs genotypes were incubated in a final volume of 50 µl containing HEPES 20 mM pH 7, NaCl 100 mM, MgCl_2_ 10 mM and a variable concentration of glutaraldehyde (0%, 0.5% and 1%) for 5 minutes at 37°C. Then, 10 µl of each sample (with or without boiling for 5 min) were resolved in a 10% SDS-PAGE gel. P-particles were further analyzed by size exclusion chromatography in a Superdex 200 column (GE Healthcare) coupled to an Äkta Purifier FPLC system (GE Healthcare). The particles were eluted in Tris-HCl 50 mM pH 7, NaCl 100 mM, 10% glycerol at 0.5 ml min^−1^. P-particles binding ability to their biological receptors was tested by Surface Plasmon Resonance (SPR) using a Biacore T100 instrument (Biacore, GE Healthcare). Human serum albumin (HSA) functionalized with the H type I antigen (Fuc(á1–2)Gal(â1–3)GlcNAc(â1–3) Gal(â1–4)(Glc)-HSA; HSA-AgH) or the Sialyl-Lewis^X^ (Neu5Ac(á2–3)Gal(â1–4)[Fuc(á1–3)]GlcNAc(â1–3)Gal(â1–4)(Glc)-HSA; HSA-SiLe^X^) antigen were purchased from Isosep AB (Uppsala, Sweden) and immobilized on the surface of CM5 chips (GE Healthcare). For immobilization the Amine Coupling Kit (GE Healthcare) was utilized and each glycoconjugate was immobilized at 500 resonance units (RU). HSA-AgH and HSA-SiLe^X^ were immobilized in channels 2 and 4 of the CM5 chips, respectively, and the channels 1 and 3 were left as references. Several fold dilutions of NoVs P-particles (300 nM to 1 nM), including repetitions and blanks from both genotypes (GI.1 and GII.4), were injected in the CM5 chip at a flow rate of 30 ìl min^−1^ in HBS-Ep+ buffer (GE Healthcare) at 25°C. The binding time was 120 seconds with dissociation times of 400 seconds. Binding and kinetics evaluations were performed with the Biacore Evaluation Software.

### Transmission electron microscopy

P-particles from genotypes GI.1 and GII.4 resuspended in PBS at 5 ìg/ml were adsorbed to electron microscopy carbon-formvar coated grids. The grids were stained with 2% phosphotungstic acid pH 6.5 and samples were observed in a Jeol GEM-1010 transmission electron microscope.

### Binding of P-particles to bacteria

Bacteria were grown overnight and washed three times with PBS before being resuspended in PBS and adjusted to an OD_550_ of 1. Normalization of the strains suspensions at the same OD but not at the same CFU ml^−1^ was carried out in order to minimize the influence of different bacterial sizes, shapes and/or the presence of cell chains of different lengths in binding results. To 200 ìl of cells, 1 ìg of P-particles from the different NoVs genogroups was added and samples were incubated for 1 h at 37°C. After this period, bacterial cells were centrifuged and washed two times with PBS to remove unbound P-particles. Finally, the bacterial pellets were resuspended in 40 ìl of Laemmli loading buffer, heated for 10 min at 100°C and 10 ìl aliquots were loaded onto 10% SDS-PAGE gels. The gels were transferred to nitrocellulose membranes (GE Healthcare) in a semi-dry western blotting apparatus. P-particles quantification was performed by chemiluminiscence with the ECL Advance Kit (GE Healthcare) by using mouse anti-6X(His) antibody (1∶10,000, Clontech) followed by horseradish peroxidase-labeled anti-mouse antibody (GE Healthcare, 1∶10,000). Each pair of interactions was assayed at least in three different experiments with bacteria coming from independent cultures. The signals in the western blot membranes were recorded with a LAS-1000 apparatus (Fuji) and quantified with the ImageGauge 4.0 software (Fuji). For immunofluorescence analysis of GI.1 P-particles bound to *L. casei* BL23 and *E. coli* Nissle 1917 bacterial cells, the bacteria were incubated with GI.1 P-particles and washed as described above. Control experiments without P-particles addition were performed. After fixation in 3% formaldehyde for 15 min the bacterial suspensions were spread on glass microscope slides and air dried. The slides were blocked for 1 h at room temperature in PBS containing 1% BSA and the P-particles were detected by incubation with mouse anti-6X(His) antibody (1∶1000, Clontech) followed by AlexaFluor488-labelled goat anti-mouse IgG antibody (1∶400, Molecular Probes). The preparations were mounted with 10 ìl of ProLong Gold Antifade Reagent (Life Technologies) and visualized with an Eclipse 90i fluorescence microscope (Nikon).

### Binding of P-particles to cultured cells

HT-29 cells (ATCC HTB-3) were seeded in DMEM (High glucose, Na-pyruvate, Gibco) supplemented with 10% (vol/vol) fetal bovine serum, 1% (vol/vol) of antibiotics (100 U ml^−1^ penicillin, 100 ìg ml^−1^ streptomycin; Gibco), 1% (vol/vol) Na-bicarbonate solution (Gibco) and 1% (vol/vol) L-glutamine 200 mM solution (Gibco) at a density of 2×10^5^ cells per cm^2^ in 96-well plates and incubated at 37°C in a CO_2_ incubator until they reached confluence (around three days of incubation). Then, they were fixed in formaldehyde 3% in PBS during 15 minutes, followed by 3 washes with PBS. Cell fixation was required in order to avoid the cells detachment that normally occurred during the prolonged incubation times and washings performed in experiments carried out with bacteria (see below). The monolayers were incubated with 100 ìl of PBS containing 10, 5, 2.5, 1.2 and 0.6 ìg ml^−1^ GI.1 and GII.4 P-particles, respectively, for 5 h at 4°C. The wells were washed with 200 ìl of PBS three times and the bound P-particles were recovered by adding 50 ìl of Laemmli loading buffer 2X per well. 30 ìl of each sample were separated in 10% SDS-PAGE gels and the His-tagged P-particles were detected by western blot as described above.

For immunofluorescence detection of P-particles bound to HT-29, cells were cultured on glass coverslips until a confluence state was reached, and incubated with GI.1 P-particles in PBS at 5 ìg ml^−1^ for 2 h at 4°C. Control experiments were performed without P-particles addition. Unbound P-particles were washed three times with PBS and P-particles retained on HT-29 cells were fixed with 3% formaldehyde. The P-particles were detected with mouse anti-6X(His) antibody (1∶1000) and AlexaFluor488-labelled goat anti-mouse IgG antibody (1∶400) after which a new fixation stage was done, followed by DAPI staining. The preparations were mounted with ProLong Gold Antifade Reagent and observed under fluorescence microscopy.

### Binding of P-particles to cultured cells in the presence of bacteria

Three types of experiments were performed with GI.1 P-particles and two different bacterial strains (*L. casei* BL23 and *E. coli* Nissle 1917) that were grown for 16 h, washed and resuspended in PBS: (i) Competitive exclusion assay. 2.5 ìg of GI.1 P-particles were mixed with 250 ìl of bacterial cells adjusted to different OD_550nm_ (1, 0.5, 0.25, 0.13 and 0.06) in PBS and immediately 100 ìl of these mixes were transferred to fixed HT-29 monolayers in duplicate wells. Incubation continued for 5 hours at 4°C after which the wells were washed 3 times with 200 ìl of PBS. (ii) Exclusion assay. HT-29 monolayers were fixed and incubated with 100 ìl of bacterial cells at OD_550_ of 1, 0.5, 0.25, 0.13 and 0.06 for 2 hours in duplicated wells. After this period, the wells were washed with PBS two times and 100 ìl of GI.1 P-particles (5 ìg ml^−1^ in PBS) were added to each well, followed by incubation for 3 hours at 4°C. Finally, the wells were washed 3 times with PBS. (iii) Displacement assay. 100 ìl of GI.1 P-particles (5 ìg ml^−1^ in PBS) were incubated in duplicated wells with fixed HT-29 cells for 2 hours. Later, the wells were washed with PBS 2 times and 100 ìl of bacterial cells at different OD_550_ in PBS were added to the wells and incubated for 3 hours at 4°C. Finally, the wells were washed 3 times with PBS. In the 3 types of assays the P-particles bound to HT-29 cells were collected with 50 ìl of Laemmli loading buffer 2X and the His-tagged P-particles were detected by western blot and quantified as described above. The experiments were repeated at least three times with independent HT-29 and bacterial cultures.

### Bacterial attachment to cultured cells

HT-29 cells were cultured and treated as described above in 96-wells plates. *L. casei* BL23 and *E. coli* Nissle 1917 bacterial cells were added to the wells at different optical densities (OD_550_ from 0.06 to 1, corresponding to 4.5×10^7^ to 7.2×10^8^ and 8.8×10^7^ to 1.4×10^9^ CFU ml^−1^ for *L. casei* BL23 and *E. coli* Nissle 1917, respectively) in 100 ìl of PBS and incubated at 4°C for 2 hours. After three washes with 200 ìl of PBS, the cell monolayers were covered with 25 ìl of 1X trypsin solution (PAA Laboratories) and incubated at 37°C for 20 min. Then, 75 ìl of PBS were added to each well and the cells and adhered bacteria were resuspended by pipetting. Appropriated dilutions were plated on MRS or LB medium for determining the numbers of adhered bacteria. Control bacterial cells at the different OD_550_ were incubated under the same conditions and treated as above with trypsin in eppendorf tubes and the CFU ml^−1^ were quantified in order to determine the amount of input cells. In order to visualize the bacteria attached to the intestinal cell line, HT-29 cells were grown on glass coverslips that were incubated as described above with bacteria at an OD_550_ of 1. After washing with PBS, the bacteria were fixed in formaldehyde 3% and the preparations were Gram stained and visualized at 100× magnification.

### Statistical analyses

The differences in binding of P-particles between different strains and conditions were evaluated with the Student T-test (SPSS 17.0 software, SPSS Inc.). In single comparisons a p value lower than 0.05 was considered statistically significant. When multiple comparisons were performed, the Bonferroni correction was applied and only differences with p values lower than 0.0045 were considered statistically significant.

## Results and Discussion

### His-tagged NoVs P-domains are structured and functional

**Figure 1:**
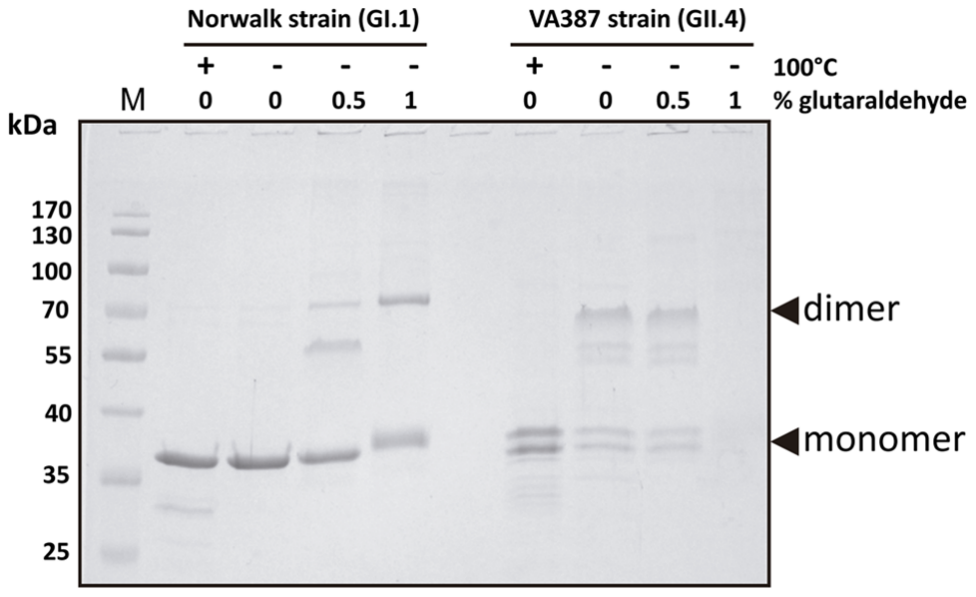
Cross-linking experiments with P-particles from GI.1 and GII.4 genotypes. Proteins were treated with glutaraldehyde and separated on SDS-PAGE gels. The purified GII.4 P domain appeared as a double band, as has been shown by others [13].

**Figure 2:**
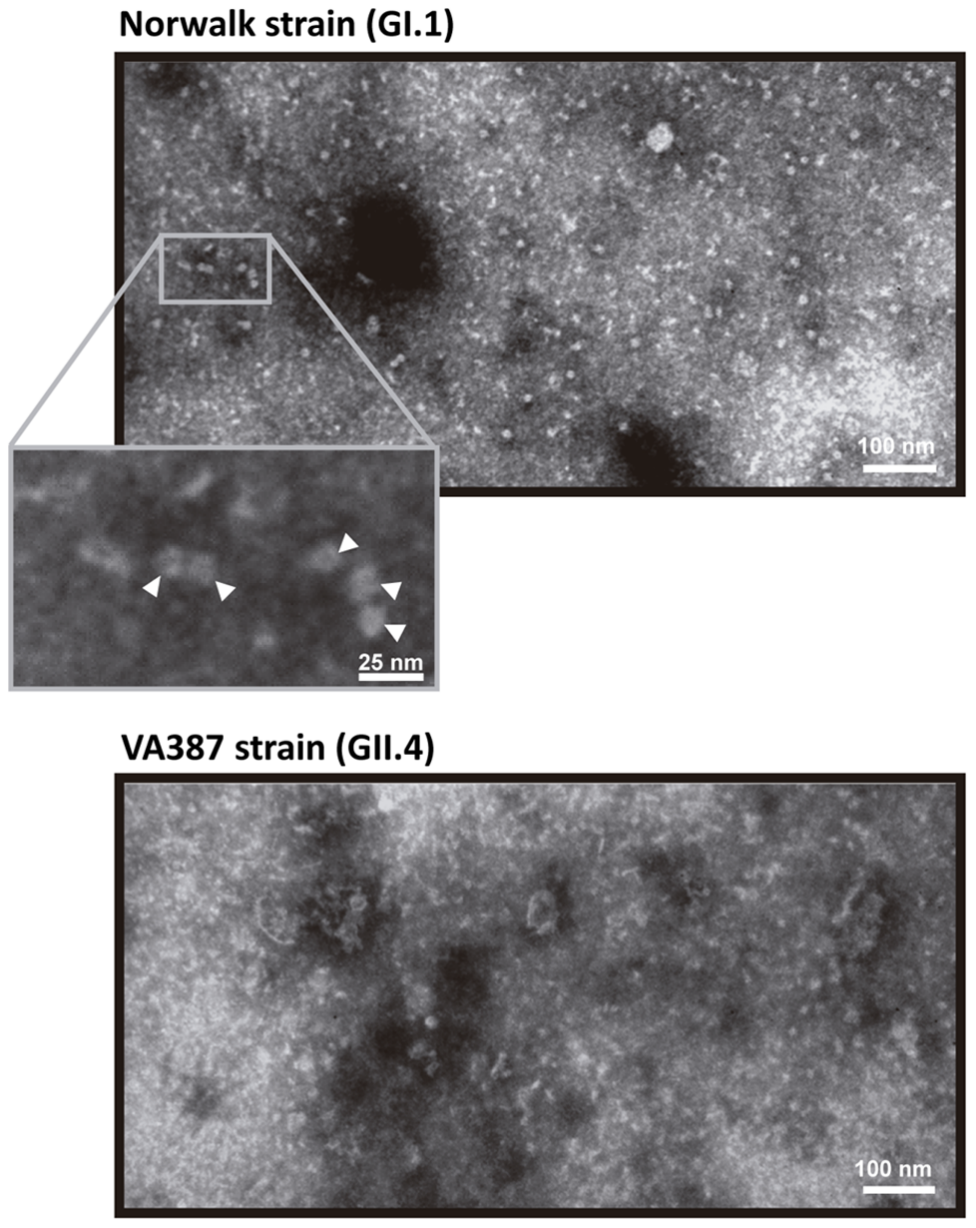
Transmission electron micrographs of GI.1 and GII.4 P-particles. The insert is an amplified section of the GI.1 micrograph highlighting the typical P-particles structures (white arrows).

In the present work we expressed NoVs P-domains with N-terminal 6X(His) tags to facilitate their purification and detection with antibodies directed against the tag. To assay if these N-terminal tagged domains maintained their biological properties we first evaluated their capacity for dimer formation [35]. The P-domains from NoVs genotypes GI.1 (Norwalk strain) and GII.4 (VA387 strain) were purified and subject to cross-linking experiments. It was observed that both domains formed at least dimers (Figure 1). These dimers were much more stable for the GII.4 genotype, since a substantial amount of the dimeric form could be already identified without the use of glutaraldehyde when samples were not boiled prior electrophoresis. For both proteins, the presence of higher molecular weight forms was also observed with increased glutaraldehyde concentrations. Treatment with a high glutaraldehyde concentration led to the disappearance of the protein bands for the GII.4 genotype, which can be explained by the formation of cross-linked P-dimers within structured P-particles [13] that are too large to enter the gel. Gel filtration analysis confirmed that the P-domains eluted in the void volume (exclusion limit of the column was 600 kDa, data not shown), although a small proportion was eluted at a calculated molecular weight of ∼350 kDa, which could be in agreement with the formation of small P-particles consisting in 12 instead of 24 P-domain monomers [36]. The formation of supramolecular structures by the GI.1 and GII.4 P-domains was further analyzed by electron microscopy, which showed the presence of structures of more than 10 nm in both genotypes (Figure 2), which was compatible with the formation of P-particles.

**Figure 3:**
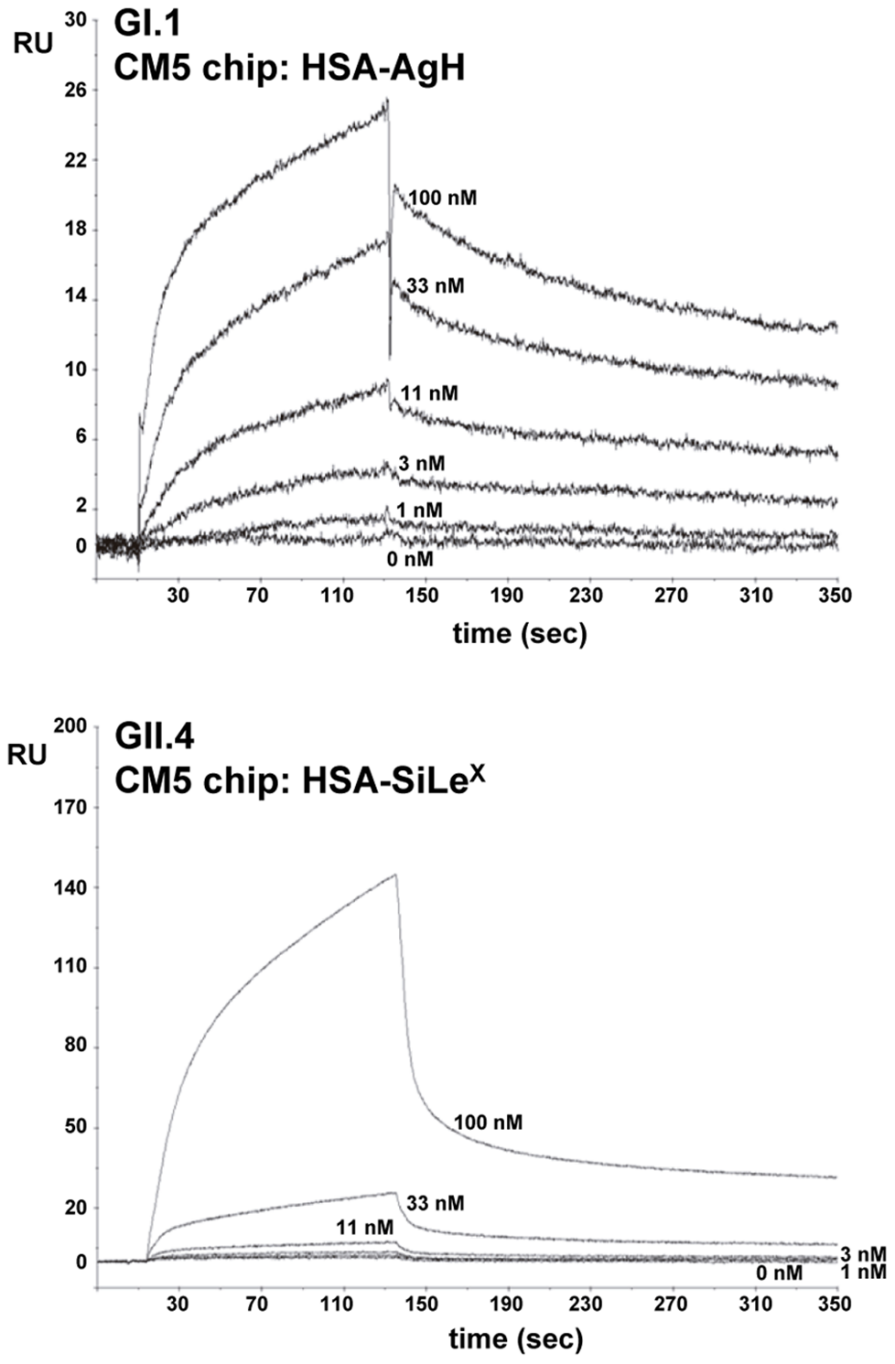
SPR sensorgrams of interaction experiments of NoVs P-particles to glycoconjugates immobilized on CM5 chips. (A) Interaction of GI.1 P-particles with HSA-AgH; (B) interaction of GII.4 P-particles with HSA-SiLe^X^. RU are resonance units. Experiments were performed with a Biacore T100 instrument.

The functionality of the P-particles was explored by determining their capacity to recognize the viral receptors. This was analyzed by SPR experiments with a Biacore T100 instrument and viral receptors immobilized on CM5 chips. The results show that each P-particle specifically recognized its receptor (Figure 3). P-particles from VA387 (GII.4) bound the SiLe^X^ glycoconjugate and those from Norwalk strain (GI.1) were specific for the antigen H glycoconjugate. Both P-particles showed very low cross-interaction with the other receptor (data not shown). The association and dissociation constants could be calculated at low protein concentrations. The determined equilibrium dissociation constants (k_D_) were 3.7 and 1.4 nM for GI.1 and GII.4 P-particles, respectively. At high protein concentrations, which promote the transition of P-domain dimers to structured P-particles [13], the presence of multiple interaction sites within each P-particle prevented the determination of kinetic parameters, as shown in similar experiments carried out with NoVs virus-like particles (VLPs) [9]. Overall, these results showed that the His-tagged P-domains or NoVs VP1 can be structured in P-particles that retain the characteristics of the viral binding for their specific host receptors. SPR can also be a valuable technique to assess viral specificity and affinity for different receptors.

### Probiotic and non-probiotic bacteria bind NoVs P-particles

**Figure 4:**
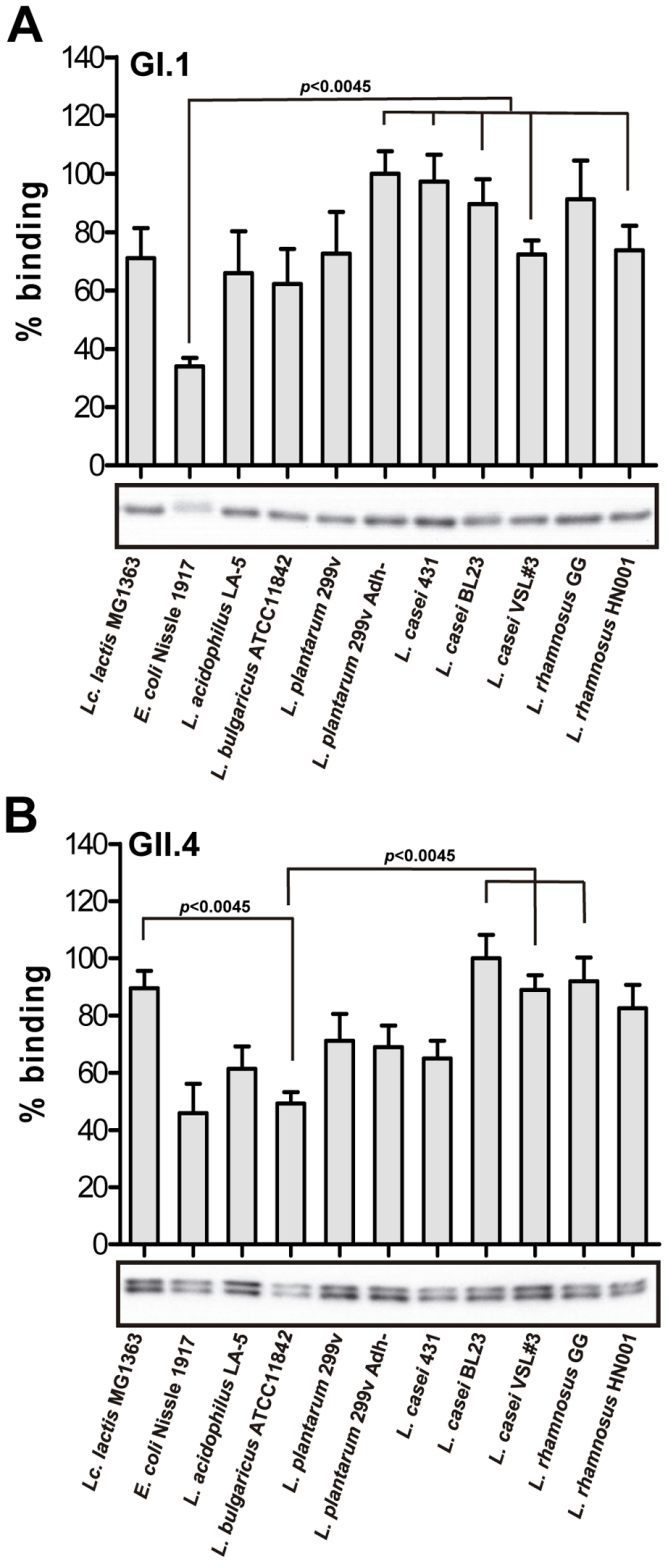
Binding of NoVs P-particles to several bacterial strains. The NoVs genotypes GI.1 (A) and GII.4 (B) are shown. The percentages of binding are referred to that of the strains with better binding. The panels below the graphs are representative western blot experiments showing the P-particles bound to bacterial cells. The experiments were repeated at least three times with independent cultures. Bars denote mean ± SE. Statistically significant differences (p<0.0045) are indicated.

Research on probiotics and viral gastroenteritis has been focused on rotaviruses, where specific probiotic strains have demonstrated their efficacy in the reduction of the duration of rotavirus diarrhea in children [17]–[20], [37], although the underlying mechanisms of these beneficial effects are mostly unknown. It has been postulated that a likely mechanism for inhibition of gastrointestinal viral infections by probiotics may consist in the direct binding of the viral particles to the bacterial surface [16], [29], similar to the capacity of some probiotics to bind heavy metals or toxins [38], promoting thus their elimination in the feces. Due to the lack of an *in vitro* model for the cultivation of NoVs and the relative difficulties in the production of VLPs in the baculovirus system, no efforts have been done until now to study the possible interactions between probiotic bacteria and NoVs. We investigated if NoVs P-particles, which share the same antigenic and HBGA-binding profile with their parental VLPs [14], could be used as a model to assess NoVs interaction with probiotics by determining the binding ability of several LAB (probiotic and non-probiotic) and of a Gram-negative probiotic strain (*E. coli* Nissle 1917). The results showed that all the assayed bacteria possessed the capacity to bind both GI.1 and GII.4 P-particles (Figure 4). As determined by quantification by western blot of P-particles retained to the bacterial surface, some strains had statistically significant higher binding abilities (Figure 4) but all of them were able to bind at least the 30% compared to the better binder for each NoVs genotype. Furthermore, the binding ability did not appear an exclusive trait of probiotics, as strains from dairy environments (e.g. *Lc. lactis* MG1363 or *L. bulgaricus* ATCC11842) also bound P-particles. This correlated with previous results which showed that *Lc. lactis* bound the rotavirus NCDV (Nebraska Calf Diarrhea Virus) with higher efficiency than other probiotic lactobacilli [29]. The isogenic strains *L. plantarum* 299v and *L. plantarum* 299v Adh- did not differ in binding to the GII.4 genotype (p = 0.86), whereas binding of the GI.1 genotype was higher for the 299v Adh- strain, although statistical significance was not achieved (p = 0.14). The two *L. rhamnosus* strains, *L. casei* BL23 and *L casei* VSL#3 were among the better binders for GI.1 and GII.4 particles. For NoV GI.1 P-particles significant differences were only found between the binding of *E. coli* Nissle 1917 and *L. plantarum* 299v Adh-, *L. casei* 431, *L. casei* BL23 and *L. rhamnosus* HN001. In the binding of NoV GII.4 significant differences were found between *L. bulgaricus* ATCC11842 and *Lc. lactis* MG1363. *L. bulgaricus* ATCC11842 also bound significantly less GII.4 P-particles than *L. casei* BL23, *L casei* VSL#3 and *L. rhamnosus* GG. In general, the Gram-negative *E. coli* Nissle 1917 strain had the poorest binding to GI.1 and GII.4 P-particles. As it is well known NoVs recognize sugar receptors in glycosylated proteins from the host cell surfaces [7]–[10]. Although we only tested one Gram-negative strain in our assays, it is reasonable to think that differences between the cell surfaces of Gram-positive versus Gram-negative bacteria are at the basis of the observed differences. Compared to Gram-negative bacteria, the Gram-positive cell wall possesses a higher content of peptidoglycan and also teichoic acids that are rich in carbohydrates and may thus have a higher potential for P-particles retention. Notwithstanding, the NoVs attachment capacity could largely depend on the strain. Thus, an intestinal isolate of the Gram-negative *Enterobacter* sp. efficiently bound NoVs VLPs from several genotypes. The binding was dependent on the presence at the bacterial surface of HBGAs-like substances of polymeric nature, probably exopolysaccharides [28]. This reinforces the idea that surface carbohydrates of bacteria are responsible for NoVs attachment.

### The effect of bacteria on the binding of P-particles to enterocyte cultures

**Figure 5:**
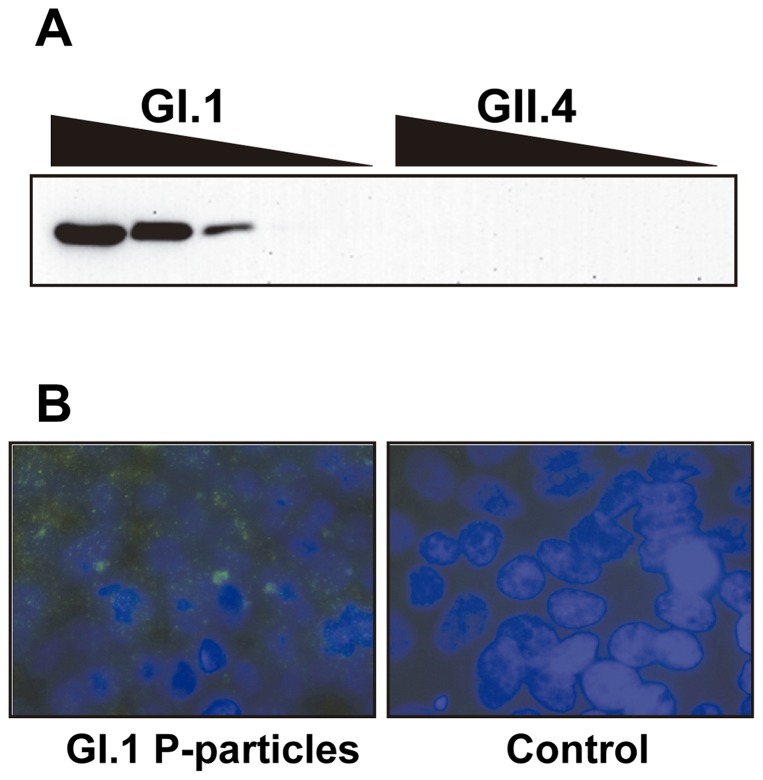
Binding of NoVs P-particles to HT-29 cells. (A) Western blot detection of P-particles bound to HT-29 cells. Cells were incubated with 10, 5, 2.5, 1.2 and 0.6 ìg ml^−1^ of GI.1 and GII.4 P-particles, respectively; (B) Immunofluorescence detection of GI.1 P-particles (5 ìg ml^−1^) bound on the surface of HT-29 cells. P-particles were detected with AlexaFluor488-conjugated antibodies (green) and HT-29 nuclei were stained with DAPI (blue).

One of the postulated mechanisms for gastrointestinal viruses inhibition by probiotics is competition and blocking of viral receptors by probiotic bacteria [16], [24], [31]. This is also supported by the fact that several lactobacilli specifically bound to different HBGAs [26]–[27]. We investigated how the presence of probiotic bacteria influences the binding of NoVs to target cells by using the P-particles and cultured intestinal epithelial cells as a model. Western blot analysis showed that P-particles from the GI.1 genotype adhered to non-differentiated human intestinal epithelial HT-29 cells in a dose-dependent manner (Figure 5A). This interaction was also confirmed by immunofluorescence detection of GI.1 P-particles bound to the cells (Figure 5B). This was in agreement with previous results which showed specific binding to this cell line of Norwalk virus VLPs [39] and indicates that the utilized GI.1 P-particles posses structural and functional similarities to their VLPs. However, GII.4 P-particles did not adhere to HT-29 cells (Figure 5A). In this regard it has been shown that differentiated Caco-2 cells were more active in binding NoVs VLPs than undifferentiated cells. [39]–[40]. Furthermore, the binding of GII.6 VLPs to Caco-2 cells revealed that only a small cell population from a Caco-2 monolayer was positive to NoV VLP binding [41]. We did not show such heterogeneity in P-particle binding in the HT-29 cells population, as binding was observed for all cells (Figure 5B). Therefore, this cell line and GI.1 P-particles were chosen for the assays for its good attachment capacity that was independent of the differentiation state [39].

**Figure 6:**
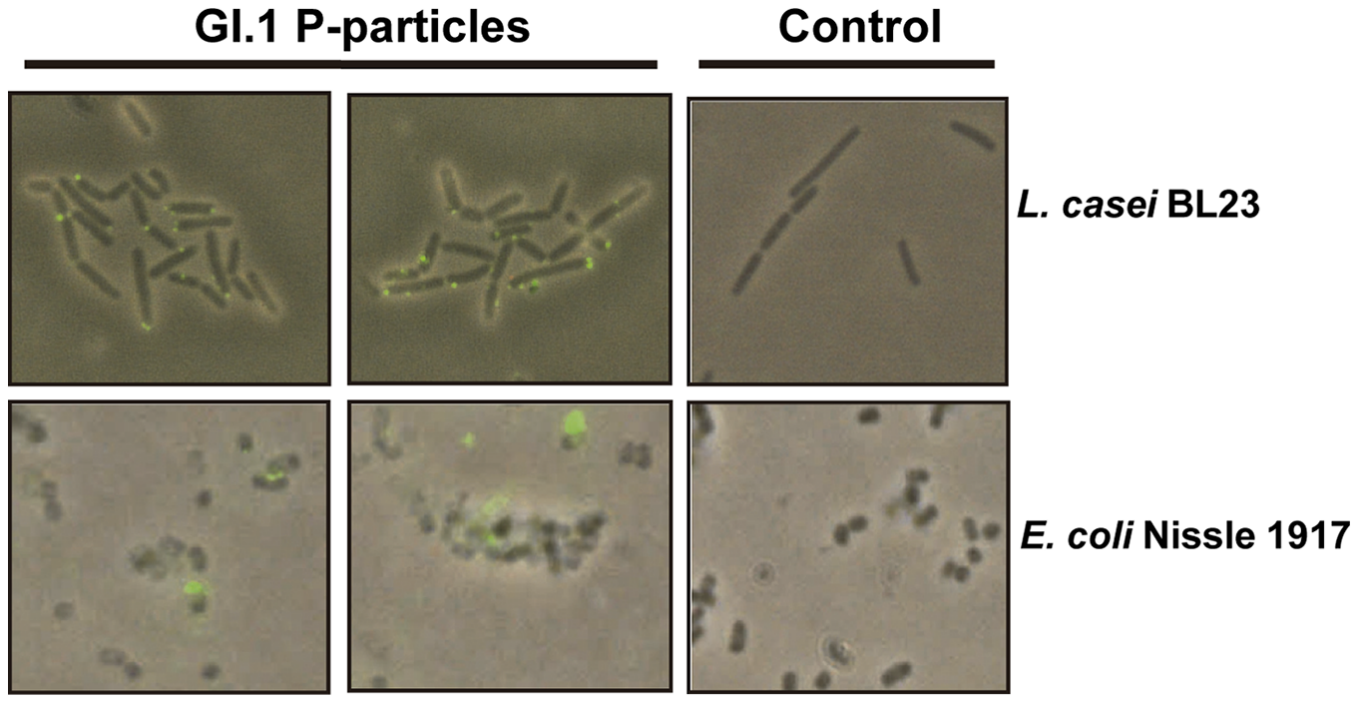
Binding of GI.1-P particles to *L. casei* BL23 and *E. coli* Nissle 1917 strains. Bacteria were incubated with GI.1 P-particles that, after washing, were detected with anti-His and anti-mouse IgG AlexaFluor488 conjugate (green). The images are a combination of phase contrast and fluorescence images. The controls are bacterial preparations incubated without P-particles.

**Figure 7:**
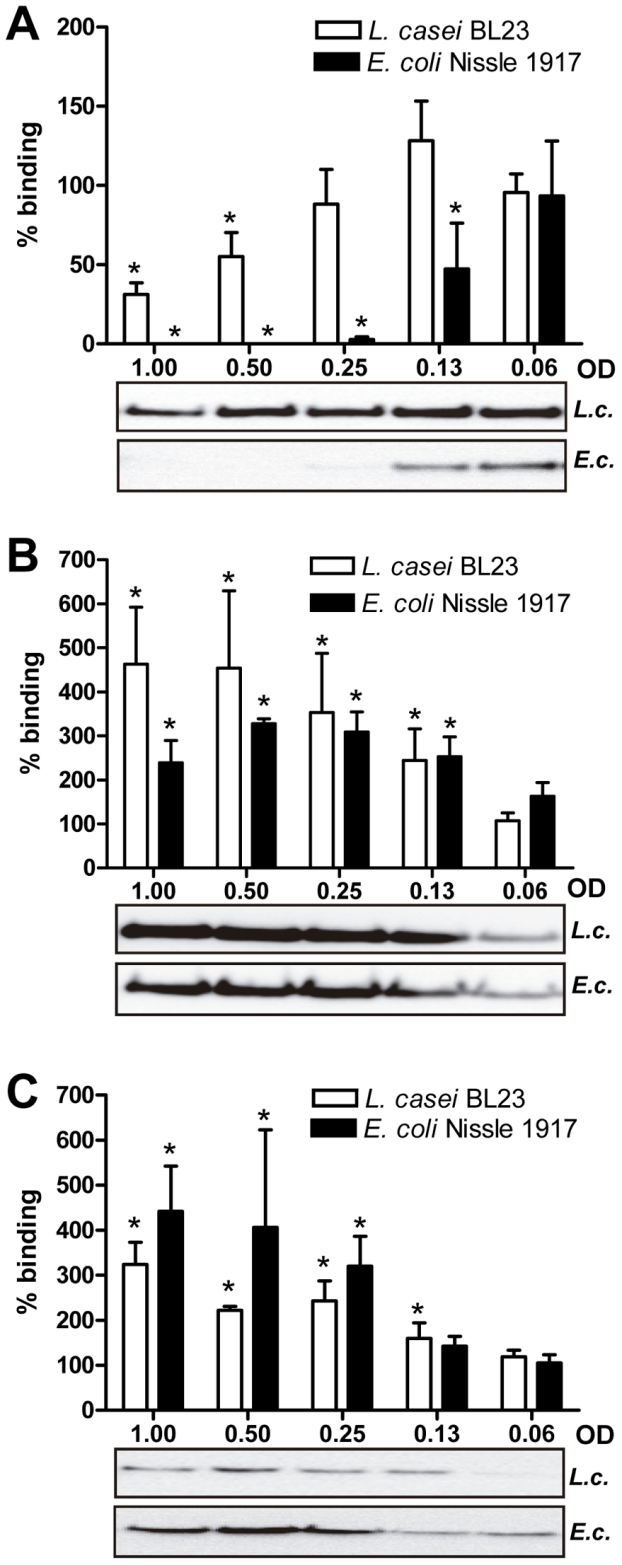
The effect of different bacteria in binding of GI.1 P-particles to HT-29 cells. HT29-monolayers were incubated with GI.1 P-particles and the viral proteins bound to the cellular surface were quantified by western blot. (A) Competitive exclusion experiments in which both the P-particles and bacteria were incubated together with HT-29 monolayers; (B) Exclusion experiments. HT-29 cells were incubated with bacteria and washed prior addition of P-particles; (C) Displacement experiments. P-particles were incubated with HT-29 monolayers that were washed prior incubation with bacterial cells. The results are expressed as percentages respect to the results obtained in the absence of bacteria. The panels below the graphs are representative western blot experiments showing the P-particles bound to HT-29 cells. *L.c.*, *L. casei* BL23; *E.c.*, *E. coli* Nissle 1917. The experiments were performed at least three times with independent bacterial and HT-29 cultures. Bars denote mean ± SE. Asterisks above the bars indicate a statistically significant difference relative to results in the absence of bacteria (p<0.05).

**Figure 8:**
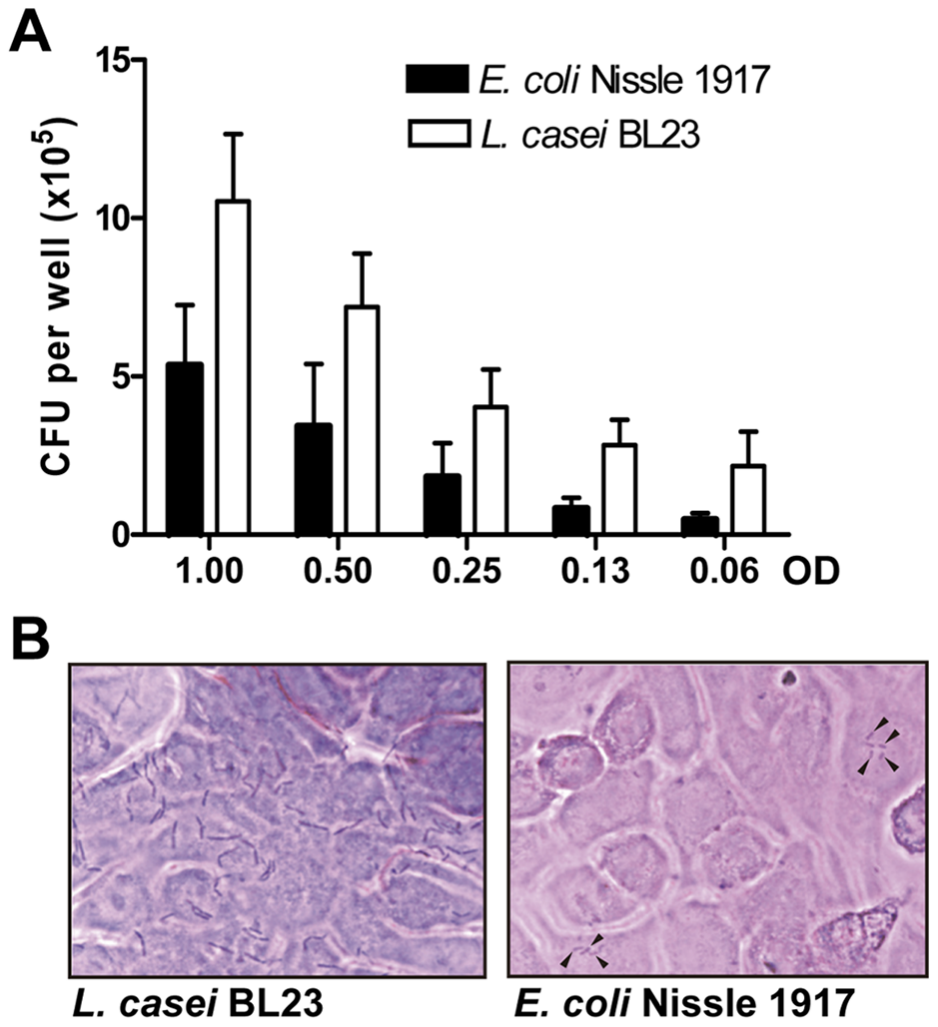
Binding of *L. casei* BL23 and *E. coli* Nissle 1917 to HT-29 cells. (A) CFUs recovered from HT-29 monolayers incubated with the bacterial strains at different OD_550_. Results are the means ± SE of three independent experiments. (B) Micrographs showing bacterial cells attached to the HT-29 monolayer. Bacteria were detected by Gram-staining. Black arrows point to adhered *E. coli* cells.

Two strains representing high (*L. casei* BL23) and low (*E. coli* Nissle 1917) GI.1 P-particles binding were selected for these experiments. In these strains GI.1 P-particles binding to the surface was observed by immunofluorescence, evidencing a preferential localization of the P-particles at the bacterial cell poles in strain BL23 (Figure 6). When competitive exclusion assays were performed (presence of bacteria in the binding assays), a negative effect on P-particle attachment to HT-29 cells was obtained (Figure 7A). In contrast to what could be predicted from the results of P-particles interaction with bacteria (Figure 4), in these assays the efficiency in blocking P-particles binding to HT-29 was superior for *E. coli* Nissle 1917 compared to *L. casei* BL23, resulting in a total inhibition of P-particles interaction at optical densities of 0.5 or 1 (Figure 7A). Also, at specific bacterial densities (OD = 0.13, Figure 7A) a positive but insignificant effect in P-particles binding to HT-29 cells was observed for the *L. casei* BL23 strain. In this line of results, when exclusion assays (incubation with bacteria followed by incubation with P-particles) or displacement assays (incubation with P-particles followed by incubation with bacteria) were performed, we came to the unexpected finding of an enhanced P-particle attachment to the monolayer surface in the presence of bacteria (up to more than 4-fold increase; Figures 7B and 7C). Differences were observed in the efficacy of the two assayed strains between the exclusion and displacement experiments, but in both cases a clear positive effect on P-particles attachment was evidenced. This implies that in this model system the bacteria would favor viral interactions with the cells. The results obtained in the displacement assays also suggest that the interaction of bacteria with cells that have already attached P-particles prevents the release of P-particles in subsequent washings. The differences observed between the competitive exclusion (Figure 7A) and the exclusion or displacement assays (Figures 7B and 7C) can be explained by several facts: when both bacteria and P-particles are in suspension (competitive exclusion experiments), interaction of P-particles with bacteria may have a strong effect in the reduction of the amount of P-particles that are available to bind to cell receptors. However, in the exclusion and displacement assays other interactions may play a role, such as the affinity of P-particles for bacteria attached to the cultured cells, which may led to higher P-particle retention on the HT-29 surface. We showed that *L. casei* BL23 and *E. coli* Nissle 1917 were indeed binding to HT-29 cells (Figure 8), although the binding was low, as has been previously reported [42]–[43]. Under our conditions a better adhesion was detected in *L. casei* BL23 compared to *E. coli* Nissle 1917. This implies that the stronger inhibition of P-particles attachment found in the competitive exclusion assays for *E. coli* Nissle 1917 may be based on additional factors besides bacterial adhesion. Similarly, *E. coli* Nissle 1917 has been shown to prevent invasion of *Salmonella*, *E. coli*, *Yersinia enterocolitica*, *Shigella flexneri*, *Legionella pneumophila* or *Listeria monocytogenes* strains in intestinal cells by a mechanism that does not involve cell or bacterial contact and depends on non-microcidal substances derived from the probiotic [43]–[44].

Studies on exclusion and displacement of bacterial pathogens by probiotics showed the efficacy of many strains in preventing pathogen adhesion to target cells [15], [45]. However, and in line to our results with NoVs P-particles, there are specific examples where the presence of probiotics enhanced pathogen attachment. Thus, *L. rhamnosus* GG enhanced the adhesion of *E. coli* O157 to human intestinal mucus by 50% in *in vitro* displacement assays [45]. Also, *L. rhamnosus* GG and an intestinal *L. rhamnosus* isolate enhanced the adhesion of *Salmonella typhimurium* ATCC14028 to the same substrate up to 300%, and this effect was related to a direct interaction of the probiotic with the pathogen [46]. Finally, two probiotic *Enterococcus faecium* strains of veterinary use improved the adhesion of *Campylobacter jejuni* to mucus up to 200%, showing that this specific combination of probiotic/pathogen may be a potential risk factor [47].

Contrarily to bacterial pathogens, research on the effect of probiotics in intestinal viruses attachment is scarce. Botiæ et al. [30] used vesicular stomatitis virus (VSV), a non-intestinal pathogen, as a model to study probiotic–virus–host interactions and showed that several probiotics reduced the infectivity of VSV up to 60% in pig intestinal epithelial cells by competition assays. Also, it was demonstrated that probiotics prevented VSV attachment by viral adsortion on their surfaces. The work presented here is to the best of our knowledge the first time that attachment assays are performed with probiotics and NoVs capsid proteins. The results obtained in this model are somehow surprising and contrary to the results obtained with VSV [30] and with specific combinations of probiotics and rotavirus in other *in vitro* models [24], [31]. However, they are in agreement to the studies described above for specific probiotic/bacterial pathogen pairs and to previous studies which have reported the positive role of feces components/intestinal microbiota in viral infection [48]. The priming of a porcine jejunum epithelial cell line with *L. acidophilus* NCFM resulted in an increased replication of porcine rotavirus [23]. The attachment of NoVs VLPs to several histo-blood antigens was also promoted by unidentified stool components [49]. This last effect was not abolished by denaturation or chelation, excluding the participation of antibodies/proteins or cations and leaving open the possibility that the microbiota, or lipids or carbohydrates derived from it, participate in the process [49]. Kuss et al. [50] demonstrated that intestinal microbiota depletion in mice reduced viral pathogenesis by reducing viral replication of two enteric viruses (poliovirus and reovirus). Incubation of poliovirus with Gram-negative or Gram-positive bacteria enhanced viral infectivity. Moreover, the presence of *Bacillus cereus* promoted a two-fold increase in the attachment of poliovirus to HeLa cells. This work also demonstrated that bacterial surface polysaccharides containing *N*-acetylglucosamine (peptidoglycan or lipopolysaccharide) interact with viral particles, promoting poliovirus attachment to host cells [50]. These observations highlight the role that intestinal microbiota may play in host susceptibility to viruses, which can result not only in reduced but also in enhanced viral infection.

In summary, the work presented here evaluates for the first time the possible interactions between one of the most important intestinal viruses producing gastroenteritis outbreaks and a panel of probiotic and non-probiotic bacteria. Although some probiotics are able to bind rotaviruses [29] and, as shown here, may have the capacity to bind NoVs, the contribution of this interaction to the reported beneficial effect on viral gastroenteritis and whether probiotics can effectively block viral attachment *in vivo* has to be carefully evaluated. The *in vitro* model presented here does not allow predicting positive or negative effects on NoVs gastroenteritis or whether specific strains may represent a risk factor, but it can serve as a first screening test for strains evaluation and for the study of the mechanisms/components involved in the probiotics-NoVs interaction. *Ex vivo* or *in vivo* animal models are needed to disclose the real role of probiotics in preventing or enhancing NoVs attachment to host cells.
